# The scent of emotions: A systematic review of human intra‐ and interspecific chemical communication of emotions

**DOI:** 10.1002/brb3.1585

**Published:** 2020-03-25

**Authors:** Elisa Calvi, Umberto Quassolo, Massimiliano Massaia, Anna Scandurra, Biagio D'Aniello, Patrizia D'Amelio

**Affiliations:** ^1^ Department of Medical Sciences University of Turin Turin Italy; ^2^ Department of Biology University of Naples “Federico II” Naples Italy; ^3^ Department of Medicine, Geriatric Medicine and Geriatric Rehabilitation CHUV Lausanne University Hospital Lausanne Switzerland

**Keywords:** behavior, body odors, chemosignals, neuroendocrinology, psychology

## Abstract

**Objective:**

The sense of olfaction has been considered of minor importance in human communication. In recent years, evidence has emerged that humans might be influenced by unconscious messages sent through chemosignals in body odors. Data concerning the ability of humans to recognize fear, maybe related to the evolutionary role of these emotions in the fight‐or‐flight reactions, are well known.

**Methods:**

To further understand the role of emotional chemosignals in mediating communication in humans and its influence on animal behaviors, we conducted a systematic literature review.

**Results:**

Chemosignals derived from axillary odors collected under a variety of emotional stimuli and sad tears in humans affect receivers' social interactions, danger detection and risk‐taking behavior, social aspects of eating, and performance under stressing conditions. In addition, beyond the fight‐or‐flight response, even the body odors of happiness can be perceived by others. Furthermore, human chemosignals can influence behaviors and stressful responses in animals, particularly dogs and horses, which may partially explain their special relationship with humans.

**Conclusion:**

Our review highlights the importance of chemosignaling in human intra‐ and interspecific interactions and suggests the need for further investigations, both in physiological conditions and in patients with psychiatric or neurodegenerative disorders.

## Graphical abstract

In recent years, evidence has emerged that humans might be influenced by unconscious messages sent through chemosignals in body odors. This is a systematic review of the studies on communication of emotions by chemosignals in humans and between humans and other species. Human chemosignals of fear and happiness can affect a variety of receivers' behaviors in humans. Furthermore, human chemosignals can influence behaviors and stressful responses in animals.

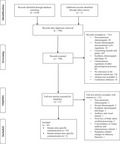

**Summations**

- Humans are able to sense and react to intraspecific chemosignals enclosed in body odors, but the exact composition of chemosignals is unknown and data on transmission of “positive emotions” trough body odors are lacking
- As data on the role of chemosignaling in demented and psychiatric patients are missing, there is high potential for further studies on emotional chemosignaling in humans
- Dogs and horses are influenced by human emotional chemosignals

**Limitations**

- Our search strategy was restricted to English‐language publications, published between January 1970 and April 2019, inaccessible or inadequately indexed reports were not taken into consideration.
- There is a considerable heterogeneity in the methodology, quality, populations, and outcomes between studies
- The number of studies providing data on chemosignaling communication between animals and humans is small

## 1 INTRODUCTION

Since the neuroanatomical studies of Paul Broca in the 19th century, the role of the olfactory system has been considered of minor relevance in humans. It is claimed that primates' evolutions have been associated with an important development of vision to the detriment of the olfaction (Liebetanz, Nitsche, Ichael, Fromm, & Reyher, 2002). The primates olfactory structures have declined over their evolution: structures as the accessory olfactory system (AOS), including the vomeronasal organ (VNO) and accessory olfactory bulb, are reduced if compared to the main olfactory system (MOS) (Heritage, 2014). This observation drives scientific efforts toward the study of the other senses, leaving the olfactory function largely unexplored. Nevertheless, it is well known that primates maintain a variety of sebaceous and apocrine skin glands (Montagna & Yun, 1972) as well as an excellent olfactory sensibility expressed as ability in discriminating odorants involved in reproductive signaling, even if compared to dogs and rats (Laska, 2000). In addition, a number of studies showed in primates the involvement of olfaction, not only in scent marking (Heymann, 2006), but also in social and sexual behaviors (Kappeler, 1998), the communication of reproductive status or the pair‐bonding (Snowdon, Ziegler, Schultz‐Darken, & Ferris, 2006).

Olfactory receptors (ORs) are 7‐transmembrane receptors activated by a G protein‐dependent pathway (Buck & Axel, 1991). Almost 400 intact OR genes have been identified in humans, a small number in comparison with dogs and rodents. Once odorant molecules bind to ORs, the signal transduction is mediated by the cilia of olfactory sensory neurons (OSNs) through the increase in intracellular cyclic adenosine monophosphate leading to neuron depolarization. OSNs converge onto glomerular structures in the olfactory bulb from which mitral cells project directly to the primary cortex, without thalamic relay, thus distinguishing the sense of olfaction from all the other human senses (Menini, 2010). Nevertheless, in recent years the involvement of the medio‐dorsal nucleus of the thalamus (MDT) in processing olfactory stimuli has been postulated (Price and Powell, s.d.) as the MDT receives inputs from all the primary olfactory areas including the piriform cortex and some secondary olfactory areas, potentially involved in olfactory stimuli processing including odor identification, discrimination, attention, and learning (Courtiol & Wilson, 2015). The detection of pheromones in humans was thought to be completely segregated by the MOS and mediated by the VNO, although its functional involvement and presence is still questioned in humans (Meredith, 2001). The VNO is a tubular structure situated in the nasal septum, part of the accessory olfactory system and specialized in detecting pheromonal involatile signals through direct physical contact (Bhatnagar & Smith, 2001). The accessory olfactory bulb, receiving inputs from vomeronasal sensory neurons axons, projects mainly to the medial and posteromedial cortical amygdala, and then to the hypothalamus, controlling reproductive and social behavior (von Campenhausen & Mori, 2000).

Nevertheless, the AOS and MOS functions are more integrated than previously thought, as both structures can respond to the same chemical stimuli and both sensory systems send projections to brain areas that are involved in mediating pheromonal responses (Brennan & Zufall, 2006).

Olfactory communication is of pivotal importance in animals' social interaction. Body odors and volatile compounds in urine, feces, or blood have been demonstrated to be a warning signal to prey species (Schauber, 2008), activating many autonomic, endocrine, and behavioral responses (Ulrich‐Lai & Herman, 2009). For example, mice smelling a mixture of pyrazine from the wolves' urine increased both vigilance behaviors and activity of the neurons in the AOS; the same substances suppress the approach of deer to feeding areas while eliciting fear responses (Osada, Miyazono, & Kashiwayanagi, 2015). Some authors hypothesized that predator odors could be detected by specific olfactory structure as MOS‐mediating responses to volatile cues (Firestein, 2001) and AOS for chemical cues or pheromones (Breer, Fleischer, & Strotmann, 2006). Specific brain areas as amygdala and hippocampus play a key role in activating autonomic and endocrinological responses (e.g., hypothalamic–pituitary–adrenal axis). Amygdala is also involved in the unconditioned fear behavior related to predator odor and in the retrieval of contextual fear memory associated with prior predator odor experiences.

It is widely recognized that humans' five senses work together in providing information and that signals received from one sense can modulate the information received from another in a multisensory way (Stein & Meredith, 1993). The relationship between visual, auditory, and somatosensory inputs, the so‐called “physical senses,” has been largely studied (Alais, Newell, & Mamassian, 2010). With regard to olfaction, we know that interaction with taste is fundamental in appetite modulation and perceptions of the foods (McCrickerd & Forde, 2016). Moreover, visual perception can affect olfactory identification (i.e., in white versus red wine identification by expert tasters as demonstrated by the study of Morrot, Brochet, & Dubourdieu (2001) and *vice versa*, modulating food‐images attractiveness, human faces pleasantness (Cook et al., 2015; Luisa Demattè, Sanabria, & Spence, 2006) or facial emotion recognition (Seubert, Gregory, Chamberland, Dessirier, & Lundström, 2014).

The sense of olfaction is unique in projecting directly to the amygdala and the orbitofrontal cortex, thus providing a close connection with the limbic system, expressly tasked with emotion processing (Hackländer, Janssen, & Bermeitinger, 2019; Krusemark, Novak, Gitelman, & Li, 2013).

A number of behavioral studies demonstrated that olfactory cues makes memories more emotional and evocative if compared to other sensory stimuli (Herz, 2016; Herz, Eliassen, Beland, & Souza, 2004). Moreover, functional magnetic resonance imaging (fMRI) studies demonstrated that memories elicited by odor perception activate specific neuroanatomical area if compared to other sensory stimuli (Herz et al., 2004).

Olfaction is also involved in odor disease avoidance: The inflammatory process leads to the release of volatile molecules in urine and feces that are recognized by conspecifics, providing information about the health status of the odor donors. The detection of sick individuals via odor cues is well known in animals and helps to avoid disease transmission inhibiting social interactions (Arakawa, Cruz, & Deak, 2011). In humans, disease‐specific (e.g., infectious or metabolic disease) volatile organic compounds have been identified (Shirasu & Touhara, 2011). Considering the dramatic role of infections in human evolution, the ability to detect olfactory cues indicating sickness could represent an adaptive survival mechanism. Some experimental studies demonstrated an unconscious ability of healthy subjects to recognize and find repulsive body odor obtained from “sick” subjects (Olsson et al., 2014); smelling these body odors activate the odor networks as shown by fMRI (Regenbogen et al., 2017). Nevertheless, many questions remain still open and literature is lacking about the neural processes underlying the ability of humans to detect sickness.

In the last decades, it has become clear that also humans have excellent olfactory abilities (McGann, 2017). The exceptional ability of humans to discriminate a big number of odorants (Bushdid, Magnasco, Vosshall, & Keller, 2014) despite the limited number of functional ORs depends on a combinatorial receptor coding scheme (Malnic, Hirono, Sato, & Buck, 1999). Scientific interest has been centered on the role of olfactory communication in shaping social interactions through molecules produced in specific emotional states (Lübke & Pause, 2015). Such molecules mediating interindividual communicative exchanges were firstly classified as pheromones and are now named chemosignals (Doty, 2010).

The question if and how humans may react to chemosignals is, indeed, challenging and not completely answered by experimental studies. Data on intraspecific communication between different species of animals (Brennan, 2010; Wyatt, 2010, 2014a, 2014b) confirm the common observation that animals communicate with each other through body odors. More surprisingly, some experimental studies suggest that also humans may be influenced in their interpersonal relationships and behaviors by the unconscious messages sent through chemosignals enclosed in body odors (de Groot, Smeets, Kaldewaij, Duijndam, & Semin, 2012).

Chemosignals are molecules excreted by animals as answer to physical distress and emotions and are able to elicit behavior or physiological responses from other animals (Petrulis, 2013). Despite this definition, until now, there is no clear evidence of which molecules are able to vehicle emotions, several molecules have been indicated as chemosignals, and these molecules have to be differentiated from odors and volatile substances (Table 1 and 2). Among these molecules, the testosterone metabolite androstadienone has been indicated as a putative chemosignal and suggested to be able to communicate dominance and social threat by several studies (Banner, Frumin, & Shamay‐Tsoory, 2018; Banner & Shamay‐Tsoory, 2018; Frey, Weyers, Pauli, & Mühlberger, 2012; Hornung, Kogler, Wolpert, Freiherr, & Derntl, 2017; Zhou et al., 2014).

**Table 1:** Differentiation between odors, volatile molecules, and pheromones

Odor	Volatile molecule	Pheromone
Blend of different moieties released in organic fluids that varies according to species, sex, age, genotype, and endocrine state and/or the property of certain substances, in very small concentrations, to stimulate chemical sense receptors.	Chemical that has a high vapor pressure at ordinary room temperature.	A chemical released by one organism that modulates the behavior or physiology of a second organism of the same species, which ranges from small, volatile molecules, and sulfated steroids to large families of proteins. Its principal properties are as follows: The synthesized molecule/combination of molecules should elicit the same response as the natural stimulus in the bioassay. It should act in this way at natural concentrations. At high concentrations, spurious results may occur as nonpheromones may stimulate receptors; For multicomponent pheromones, experiments should demonstrate that all compounds in the combination are necessary and sufficient to elicit the full response; Only this molecule or the proposed combination of molecules elicits the effect (unlike other similar molecules or combinations that the animal would normally encounter); There should be a credible pathway for the pheromone signal to have evolved by direct or kin selection. In evolutionary terms, to be a signal, both the emission and reception of the pheromone signal should have evolved for a particular function.

**Table 2:** List of putative chemical messenger molecules relevant for mammals

Molecule	Supposed function	Species and secretion organ	Reference
5α‐androst‐16‐en‐3‐one	Reduction of the threshold for pressure‐induced lordosis in female pigs	Domestic pig, male salivary glands	Melrose, Reed, and Patterson (1971)
Male‐enriched 2‐(*sec*‐butyl)‐dihydrothiazole	Promotion of estrous synchronization in group‐housed females (Whitten effect) and acceleration of the onset of puberty in juvenile females (Vandenbergh effect)	Mouse, male urine	Jemiolo, Harvey, and Novotny (1986)
Dehydro‐*exo*‐brevicomin	Promotion of estrous synchronization in group‐housed females (Whitten effect) and acceleration of the onset of puberty in juvenile females (Vandenbergh effect)	Mouse, male urine	Novotny, Ma, Wiesler, and Zidek (1999)
Female‐enriched 2,5‐dimethylpyrazine	Suppression of female estrous	Mouse, female urine	Novotny, Jemiolo, Harvey, Wiesler, and Marchlewska‐Koj (1986)
2‐heptanone	Promotion of female estrous	Mouse, female urine	Jemiolo, Andreolini, Xie, Wiesler, and Novotny (1989)
MUPs (major urinary proteins)	Acceleration of puberty onset	Mouse, male urine	Mucignat‐Caretta, Caretta, and Cavaggioni (1995)
2‐methylbut‐2‐enal	Induction of an innate suckling response in neonates that have not nursed previously	Rabbit, female milk	Schaal et al. (2003)
Dodecyl propionate	Stimulation of maternal grooming	Mouse, preputial gland of neonatal rat	Brouette‐Lahlou, Godinot, and Vernet‐Maury (1999)
Salivary ABP (androgen‐binding protein)	Promotion of sexual isolation	Mouse, male salivary glands	Laukaitis, Critser, and Karn (1997)
2‐(*sec*‐butyl)‐dihydrothiazole and dehydro‐*exo*‐brevicomin	Promotion of intermale aggression (in addition to the aforementioned effects on female mice)	Mouse, male urine	Novotny, Harvey, Jemiolo, and Alberts (1985)
ESP1 (exocrine gland–secreting peptide 1)	Induction of stereotyped lordosis responses in females	Mouse, male tears	Haga et al. (2010). Knockout of V2Rp5 abolishes behavioral responses to the mouse sex pheromone ESP1.
Darcin (a nonvolatile MUP)	Determination of unconditioned attractive properties of male's urine to female mice	Mouse, male urine	Roberts, Simpson, Armstrong, Davidson, and Robertson (2010)
Aphrodisin (a lipocalin)	Induction of male sexual behavior	Hamster, female vaginal fluid	Briand, Trotier, and Pernollet (2004)
2‐(*sec*‐butyl)‐dihydrothiazole	Putative alarm pheromone (in addition to aforementioned functions)	Mouse, male urine	Brechbuhl et al. (2013)

In recent years, the involvement of chemosignals on species‐specific communication of stable features such as age, gender, kin recognition, fertility, and reproductive behavior has been extensively studied (Gildersleeve, Haselton, Larson, & Pillsworth, 2012; Jones, Hahn, & DeBruine, 2019; Marazziti et al., 2011; Mitro, Gordon, Olsson, & Lundström, 2012; Pause, 2004b; Penn et al., 2007; Weisfeld, Czilli, Phillips, Gall, & Lichtman, 2003).

In addition, research on chemosignaling is focusing on the transmission of emotional states.

Preliminary studies investigated the involvement of chemosignals in conveying emotional states from “a sender” to “a receiver.” In 2000, Chen and Haviland‐Jones were able to demonstrate for the first time that human subjects can recognize the emotion of another human subject by sniffing odors collected by axillary pads (Chen & Haviland‐Jones, 2000). In the following years, a number of further evidences confirmed that human body odors vary according to emotional states of the donors and that these changes can be perceived by receivers (Pause, 2004a; Pause, Adolph, Prehn‐Kristensen, & Ferstl, 2009; Prehn, Ohrt, Sojka, Ferstl, & Pause, 2006).

The majority of research on communication via human body odors has focused on the transmission of the so‐called “negative emotions” (i.e., fear, stress or anxiety; de Groot & Smeets, 2017), based on the evolutionary significance of potential activation of adrenergic‐mediated stress response system. In subsequent studies, similar results have been obtained with “positive emotions” as happiness or sexual arousal (Iversen, Ptito, Møller, & Kupers, 2015; Zhou & Chen, 2011; Zhou, Hou, Zhou, & Chen, 2011) showing the complexity of chemosignaling in human's communication.

Olfactory dysfunction is an early feature of Alzheimer disease (AD; Doty & Hawkes, 2019; Mesholam, Moberg, Mahr, & Doty, 1998). Neurofibrillary tangles early accumulate in the key areas for olfactory function in AD (Kovács, Cairns, & Lantos, 1999; Ohm & Braak, 1987), and neuroimaging studies demonstrate atrophy in the primary olfactory cortex and hippocampus in AD patients (Kotecha et al., 2018; Vasavada et al., 2015). Interestingly, impaired ability to identify different odors seems to predict the progression of cognitive decline in subjects with mild cognitive impairment (Devanand et al., 2000). Limited evidences suggested that olfactory dysfunction might be useful to differentiate AD from another type of dementia (Park, Lee, Lee, & Kim, 2018).

Also in Parkinson's disease, the olfactory dysfunction plays a key role in the diagnosis, as its evaluation is included in the diagnostic course, in particular in distinguishing Parkinson's disease from other parkinsonian syndromes (Suchowersky et al., 2006). In Parkinson's disease, olfactory impairment appears years before the clinical manifestation of the disease, remains stable over time, and affects more than 90% of patients (Doty, 2012). Moreover, in longitudinal studies olfactory impairment can predict the rate of evolution toward dementia (Baba et al., 2012).

Recent data suggest that humans' chemosignals could also be perceived by other species as dogs and horses (D'Aniello, Semin, Alterisio, Aria, & Scandurra, 2018; Lanata et al., 2018; Siniscalchi, d'Ingeo, & Quaranta, 2016). These findings open a new field of investigation, suggesting a deeper interpretation of the relationship between pets and their owners. In particular, they may furnish a completely new interpretation on the effectiveness of pet therapy for cognitive impaired patients (Charry‐Sánchez, Pradilla, & Talero‐Gutiérrez, 2018; Hu, Zhang, Leng, Li, & Chen, 2018; Majić, Gutzmann, Heinz, Lang, & Rapp, 2013; Wesenberg, Mueller, Nestmann, & Holthoff‐Detto, 2019; Yakimicki, Edwards, Richards, & Beck, 2019), rising the challenging hypothesis that the benefit of pet therapy relies on a deep interspecific communication beyond rationality and social conventions.

Nevertheless, many questions remain unanswered: Little is known about the brain areas involved in the recognition of the emotions transmitted through chemosignals, as well as the consequences of neurodegenerative or psychiatric pathologies on the ability to recognize the chemical messages. Furthermore, whether chemosignals are recognized through the primary olfactory system or through the VNO in humans remains controversial (D'Aniello, Semin, Scandurra, & Pinelli, 2017; Meredith, 2001) and the identification of active compounds involved in chemosignaling is far from completion. As geriatricians, we are particularly interested in understanding the different reactions of cognitive impaired patients to their professional and familiar caregivers' chemosignals (Rippon et al., 2019).

Here, we systematically review the studies on the communication of emotions by chemosignals in humans and between humans and other species. The understanding of emotional communication through chemosignals will increase our understanding of intraspecific and interspecific communications.

## 2 MATERIALS AND METHODS

### 2.1 Eligibility criteria

Inclusion criteria were based on the Participants, Intervention, Comparator, Outcomes, and Study design, the PICO model was built as follows:

*Participants*: We included studies investigating the effects of human‐derived emotional chemosignals on human and animal receivers.

*Interventions*: We included only studies analyzing the responses to emotional stimuli derived by body odors collected from a sender under an emotional condition. Studies with synthetic substances or hormonal stimuli were excluded.

*Comparator*: A control stimulus had to be presented to the receiver and included body odors obtained during exercise or after a neutral stimulus, unused sweat pads, or saline solutions.

*Outcomes*: We included studies investigating the ability of an emotional body odor to elicit the same emotion in the sender as compared to a control stimulus. Measures could be fMRI, facial electromyography (EMG), skin conductance response (SCR), electroencephalography (EEG), cardiac activity or cognitive, affective, behavioral, or perceptual tasks.

*Study design*: We included English‐language and peer‐reviewed studies with no limitations due to study type or publication date.

### 2.2 Information source

This systematic review was performed according to the Preferred Reporting Items for Systematic Reviews and Meta‐analyses (PRISMA) checklist from January 1970 to April 2019.

The search strategy was conducted to find relevant studies from the MEDLINE, EMBASE, Cochrane Library, and PsychINFO databases.

A manual search of these articles' reference lists was performed to capture additional articles for consideration; this search allowed us to find one article from Kamiloğlu, Smeets, de Groot, and Semin (2018).

### 2.3 Search strategy

The search evaluated articles using the search terms:

1. Fear
2. Emotions
3. Happiness
4. Anxiety
5. Stress
6. Disgust
7. 1 or 2 or 3 or 4 or 5 or 6
8. Chemosignaling
9. Chemosignals
10. Body odors
11. Scent
12. Chemosensory signals
13. Apocrine sweat
14. Chemosensory
15. 8 or 9 or 10 or 11 or 12 or 13 or 14
16. 7 and 15

### 2.4 Study selection

Two experienced reviewers (EC and UQ) identified all studies meeting the inclusion criteria to be included for the full review. Each reviewer independently selected studies for inclusion in the review, and discrepancies were resolved by mutual consensus.

### 2.5 Data extraction and analysis

**Figure 1:**
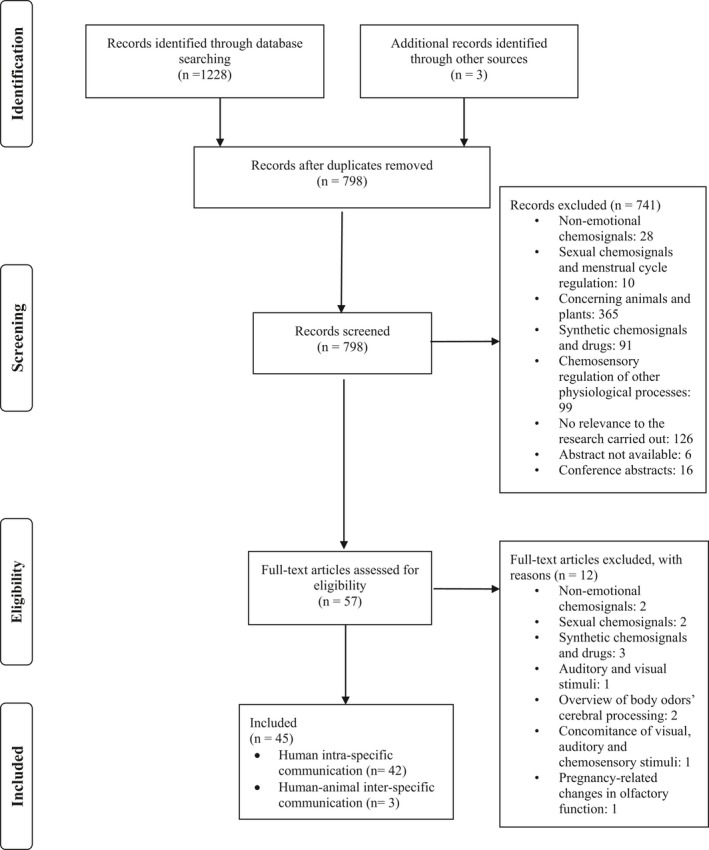
Flow diagram of the analysis of the literature

This search query returned 451 (PubMed) + 692 (EMBASE) + 11 (PsychINFO) + 74 (Cochrane) articles for review. After removing duplicates, we excluded 741 articles (Figure 1). Fifty‐seven articles were reviewed in full text by the authors and considered for evaluation. Selected articles for review were published between 2000 and 2018.

We were able to find on the Web two relevant studies as unpublished dissertation; however, we decided to exclude those studies from this review as they were not peer‐reviewed (Hatcher, s.d.; Owen, s.d.).

Twelve articles were excluded after reading the full text as they were considered nonpertinent. Based on the full‐text review, forty‐five articles were selected for full‐text, in‐depth review (Table 3). A flow diagram of the selection procedure is included in Figure 1.

**Table 3:** List of human intraspecific and human–animal interspecific communication chemosignaling studies

Emotion	Vehicle	Emotional source	Control	Assessment	Senders	Receivers	Olfactory function	Stimulus presentation	Main Outcome	Reference
Aggression	Axillary SE	Boxing session	Ergometer training	State aggression version of the STAXI questionnaire	16‐M	10‐M, 12‐F	MONEX‐40	Cellulose filter mask	Emotion recognition task, emotional stroop task	Mutic et al. (2016)
Aggression	Axillary SE	Mathematical problems with time constraint and negative feedback followed by boxing session	Mathematical problems without time constraint followed by hand‐bike training	100‐mm VAS	16‐M	12‐M, 11‐F	MONEX‐40	Cotton pads in filter masks under the participants' noses	fMRI	Mutic et al. (2017)
Anxiety	Axillary SE	Oral exam	Ergometer training	SAM	20‐M	40‐F	Three alternative forced‐choice test	Olfactometer	Startle Reflex and EEG	Adolph et al. (2013)
Anxiety	Axillary SE	High rope course	Ergometer training	Spielberger's STAI	13‐M	20‐F	Sniffin' Sticks test	Odorless teabags attached under participants' nostrils with odorless tape	Self‐Report	Albrecht et al. (2011)
Anxiety	Axillary SE	High rope course	Ergometer training	Spielberger's STAI	21‐M	14‐M, 16‐F	Sniffin' Sticks test	Odorless teabags attached under nostrils	Risk Game	Haegler et al. (2010)
Anxiety	Axillary SE	Oral exam	Ergometer training	/	28‐F	10‐F	PEA identification	Olfactometer	Startle Reflex	Lübke et al. (2017)
Anxiety	Axillary SE	Oral exam	Ergometer training	Questionnaires	12‐M	8‐M, 8‐F	/	Olfactometer	Priming	Pause (2004a,b)
Anxiety	Axillary SE	Oral exam	Ergometer training	Salivary cortisol and testosterone samples, SAM	28‐M, 21‐F	8‐M non‐SA, 8‐F non‐SA, 8‐M SA, 8‐F SA	Self‐reported	Olfactometer	Startle Reflex	Pause et al. (2009)
Anxiety	Axillary SE	Oral exam	Ergometer training	/	28‐M, 21‐F	16‐M non‐SA, 12‐F non‐SA, 8‐M SA, 8‐F SA	PEA identification	Olfactometer	EEG	Pause et al. (2010)
Anxiety	Axillary SE	Oral exam	Ergometer training	Questionnaires	12‐M	4‐M, 3‐F	Self‐reported	Olfactometer	Startle Reflex	Prehn et al. (2006)
Anxiety	Axillary SE	Oral exam	Ergometer training	Salivary cortisol and testosterone samples, SAM	28‐M, 21‐F	14‐M, 14‐F	Self‐reported	Olfactometer	fMRI	Prehn‐Kristensen et al. (2009)
Anxiety	Axillary SE	Oral exam	Regular class	Spielberger's STAI, 100‐mm VAS	6‐F	46‐F	Short version of the Sniffin' Sticks test	Olfactometer	Categorizing the emotion of a face	Rocha et al. (2018)
Anxiety	Axillary SE	3‐hr clinical session	3‐hr lecture	/	7‐M, 17‐F	7‐M, 17‐F	Screening questionnaire	Phantom patient wearing used cotton t‐shirts	Dental performance	Singh et al. (2018)
Anxiety	Axillary SE	Oral exam	Stationary cycling	SAM, VAS	10‐M	14‐M, 10‐F	MONEX‐40	Olfactometer	fMRI	Wudarczyk et al. (2015)
Anxiety	Axillary SE	Oral exam	Ergometer training	Salivary cortisol samples, Questionnaire	10‐M	14‐M, 10‐F	MONEX‐40	Odorless teabags attached under participants' nostrils with odorless tape	fMRI	Wudarczyk et al. (2016)
Anxiety	Axillary SE	High rope course	Ergometer training	Spielberger's STAI	21‐M	15‐M	Sniffin' Sticks test	Vial (placed 2 cm below the participant's nose)	Face rating	Zernecke et al. (2011)
Disgust	Axillary SE	Disgusting videos	Neutral videos	/	14‐M	16‐F	Self‐reported, clinical visit	Olfactometer	Forced‐Choice Task and fMRI	Zheng et al. (2018)
Disgust, Fear	Axillary SE	Horror or disgusting videos	Unused cotton pads	Spielberger's STAI and 7‐point Likert scales	10‐M	36‐F	Sniffin' Sticks test	Vial (placed 2 cm below the participant's nose)	EMG	de Groot et al. (2012)
Disgust, Fear	Axillary SE	Horror or disgusting videos	Neutral videos	2 separate 7‐point Likert scales, Portuguese version of PANAS	10‐M, 10‐F	37‐M, 32‐F	Sniffin' Sticks test	Polypropylene jars	3 ECG electrodes to evaluate cardiac activity	Ferreira et al. (2018)
Disgust, Fear, Happiness, Sexual arousal	Axillary SE	Horror or comical or disgusting or erotic videos	Unused cotton pads	Heart rate during watching videos, 7‐point Likert scale at the end of the videos	15‐M, 15‐F	7‐M CB, 7‐F CB, 8‐M non‐CB, 6‐F non‐CB	MONEX‐40, Sniffin' Sticks Battery	Polypropylene jars	Identification	Iversen et al. (2015)
Fear	Axillary SE	Horror videos	Neutral videos	Salivary cortisol samples, Spielberger's STAI	42‐F	62‐F	Screening questionnaire	Plastic bottles	Odor rating	Ackerl et al. (2002)
Fear	Axillary SE	Horror videos	Neutral videos	100‐mm VAS, hidden video camera	4‐M, 3‐F	50‐F	Self‐reported	Band‐aid attached at the philtrum just below the nostrils	Cognitive task	Chen et al. (2006)
Fear	Axillary SE	Horror videos	Neutral videos	7‐point Likert scales	13‐M, 13‐F	26‐M, 26‐F	PEA identification, Sniffin' Sticks test	Vial (placed 2 cm below the participant's nose)	EMG	de Groot et al. (2014)
Fear	Axillary SE	Horror videos	Neutral videos	7‐point Likert scales	8‐M	30‐F	Sniffin' Sticks test	Vial (placed 2 cm below the participant's nose)	EMG; Chinese symbol task	de Groot et al. (2014)
Fear	Axillary SE	Skydiving	Nonstressed outdoor activity	Salivary cortisol sampling, Questionnaire	16‐M	33‐M ASD, 81‐M TD, 2‐F ASD, 2‐F TD	Screening questionnaire	Glass jar covered by a cap with an air filter, inhalation mask and a one‐way flap valve	Perception task	Endevelt‐Shapira et al. (2018)
Fear	Axillary SE	Skydiving	Treadmill exercise	Salivary cortisol, Spielberger's STAI	20‐M, 20‐F	8‐M, 8‐F	Self‐reported	Olfactometer	fMRI and perception task	Mujica‐Parodi et al. (2009)
Fear	Axillary SE	Skydiving	Treadmill exercise	Salivary cortisol samples, Spielberger's STAI	20‐M, 20‐F	8‐M, 8‐F	/	Olfactometer	fMRI	Radulescu and Mujica‐Parodi (2013)
Fear	Axillary SE	Skydiving	Treadmill exercise	Salivary cortisol samples and self‐reported state of anxiety	64‐M	6‐M, 8‐F	/	Olfactometer	EEG	Rubin et al. (2012)
Fear, Happiness	Axillary SE	Horror or comical videos	Unused cotton pads	7‐point Likert scales	11‐M, 14‐F	37‐M, 40‐F	/	Glass bottles	Identification	Chen and Haviland‐Jones (2000)
Fear, Happiness	Axillary SE	Horror or comical videos	Unused sweat pads	Spielberger's STAI	8‐M	17‐M pet dogs, 23‐F pet dogs	/	Odor dispenser in the room	Dogs' behavior, stress and heart rate indicators	D'Aniello et al. (2017)
Fear, Happiness	Axillary SE	Horror or comical videos	Neutral videos	7‐point Likert scales	9‐M	36‐F	Sniffin' Sticks test	Vial (placed 2 cm below the participant's nose)	EMG	Groot, Smeets, Rowson, et al. (2015)
Fear, Happiness	Axillary SE	Horror or comical videos	Neutral videos	16 items from the affective circumplex complemented by 4 remaining discrete emotion terms. Core affect measured on a two‐dimensional affect grid	24‐M Caucasian	48‐F Caucasian, 48‐F eastern Asian	Sniffin' Sticks test	Polypropylene jars	EMG and continuous flash suppression techniques to measure unconscious emotions	de Groot et al. (2018)
Fear, Happiness	Axillary SE	Horror or comical videos	/	/	14‐M	20‐M, 41‐F	/	Unused pads	Identification	Haviland‐jones et al. (2016)
Fear, Happiness	Axillary SE	Horror or comical videos	Neutral videos	7‐point Likert scales	12‐M	24‐F	Identification of 3 different odors	Polypropylene jars	2‐alternative forced‐choice reminder task; EMG; reaction times (Rts)	Kamiloğlu et al. (2018)
Fear, Happiness	Axillary SE	Horror or comical videos	/	Spielberger's STAI	8‐M	7‐M horses	/	Test tube with cotton swab soaked with odor	Autonomic Nervous System activity	Lanata et al. (2018)
Fear, Happiness	Axillary SE	Horror or comical videos	Running, unused sweat pads	Five‐point VAS, heart rate	4‐M	11‐M pet dogs, 20‐F pet dogs	/	Vial	Dogs' behavior, stress and heart rate indicators	Siniscalchi et al. (2016)
Fear, Happiness	Axillary SE	Horror or comical videos	Neutral videos	100‐mm VAS	8‐M	48‐F (1° experiment), 16‐F (2° experiment)	Sniffin' Sticks test	Band‐aid attached at the philtrum just below the nostrils	Perception task	Zhou and Chen (2009)
Fear, Happiness, Sexual arousal	Axillary SE	Horror or comical or erotic videos	Neutral videos	100‐mm VAS	20‐M, 20‐F	20‐M, 20‐F	PEA identification; SIT	Vial (placed 2 cm below the participant's nose)	Emotion Detection Task; 7‐point Likert scale	Zhou and Chen (2011)
Happiness	Axillary SE	Sport competition	Running	Salivary cortisol and testosterone samples	6‐M	9‐M, 9‐F	PEA identification	Olfactometer	SCR	Adolph et al. (2010)
Psychosocial stress	Axillary SE	TSST	Stationary cycling	Mood ratings questionnaire	44‐F	48‐M, 72‐F	Self‐reported	Glass bottles	Rating person	Dalton et al. (2013)
Psychosocial stress	Axillary SE	Anticipatory stage of TSST	Neutral videos	Heart rate during watching videos, Salivary cortisol samples	8‐M	31‐F	Sniffin' Sticks test	Vial (placed 2 cm below the participant's nose)	EMG, facial expression classification task	Groot, Smeets, Rowson, et al. (2015)
Psychosocial stress	Axillary SE, artificial odors	TSST	Ergometer training	SAM	7‐M PD, 6‐F PD, 7‐M non‐PD, 6‐F non‐PD	13‐M or F PD with/without agoraphobia, 13‐M or F non‐PD	Sniffin' Sticks test	Intranasal Teflon™ tubing	fMRI	Wintermann et al. (2013)
Sadness	Female tears	Sad videos	Saline solution	/	2‐F	24‐M	/	Band‐aid attached at the philtrum just below the nostrils	fMRI	Gelstein et al. (2011)
Sadness	M fasting and postprandial plasma, F tears	Sad videos	Saline solution	/	20‐M, 4‐F	20‐M	/	Band‐aid attached at the philtrum just below the nostrils	Appetite assessment by a VAS	Oh et al. (2012)
Sexual arousal	Axillary SE, Androstadienone	Erotic videos	Neutral videos	Skin Conductance	6‐M	19‐F	PEA identification, Sniffin' Sticks test	Olfactometer	fMRI	Zhou et al. (2011)

Abbreviations: ASD, autism spectrum disorder; CB, congenitally blind; F, female; M, male; PANAS, positive and negative affective schedule; PD, panic disorder; PEA, phenyl ethyl alcohol; R, receiver; S, sender; SA, socially anxious; SAM, self‐assessment manikin; SCR, skin conductance response; SE, sweat extracts; STAI, state‐trait anxiety inventory; STAXI, state‐trait anger expression inventory; TD, typically developed; TSST, Trier social stress test; VAS, visual analogue scale.

The following variables were extracted from each study: year of publication, chemosignal type, emotion induction, odor control condition, assessment of induced emotion, male/female senders and receivers, olfactory function assessment, stimuli collection material, stimuli presentation, main outcome.

Data were collected using Microsoft Excel (version 16.11).

This study does not contain any studies with human participants or animals performed by any of the authors. For this type of study, formal consent is not required.

## 3 RESULTS

The studies analyzed were highly heterogeneous in methodology: They differed in the stimulus chosen (sweat or tears); in the method used for the induction of emotional response in the donors (ranging between watching different kinds of videos, to extreme sports experience); in the kind of emotion evaluated; in the subjects enrolled as donors or receivers, differences in subjects included age, sex, and sexual orientation; in the main outcomes and the methods of measurement. Table 3 describes the key characteristics of the studies included in this review.

### 3.1 Intraspecific communication

Forty‐two studies investigated intraspecies chemosignals communication in humans. Among these, in 40 studies chemosignals derived from axillary sweat extracts from a total of 568 male and 327 female donors; in the remaining two studies, chemosignals derived from sad tears from a total of 6 female donors (Gelstein et al., 2011; Oh, Kim, Park, & Cho, 2012). All donors were healthy adults (minimum and maximum age of 18 and 50 years, respectively).

In one article, donors were partners of female receivers (Zhou & Chen, 2011).

In 16 studies (Albrecht et al., 2011; Ferreira, Parma, Alho, Silva, & Soares, 2018; de Groot, Semin, & Smeets, 2014a, 2014b; de Groot et al., 2012; Groot, Smeets, Rowson, et al., 2015; de Groot, Smeets, & Semin, 2015; Haegler et al., 2010; Mutic, Parma, Brünner, & Freiherr, 2016; Rocha, Parma, Lundström, & Soares, 2018; Wudarczyk et al., 2015, 2016; Zernecke et al., 2011; Zheng et al., 2018; Zhou & Chen, 2011; Zhou et al., 2011), homosexual donors were excluded, as female perceives sweat from heterosexual donors differently than homosexual male sweat (Martins et al., 2005). In order to increase sensibility to emotional signals in receivers of the opposite sex (Martins et al., 2005), in 10 studies only heterosexual receivers were selected (Albrecht et al., 2011; Ferreira et al., 2018; de Groot et al., 2012; Groot, Smeets, Rowson, et al., 2015; Groot, Smeets, & Semin, 2015; Mutic, Brünner, Rodriguez‐Raecke, Wiesmann, & Freiherr, 2017; Mutic et al., 2016; Rocha et al., 2018; Zheng et al., 2018; Zhou & Chen, 2011), while in the other studies there is no mention of sexual orientation of the receivers.

Odor stimuli were collected on sterile absorbent pads, plastic vials, polypropylene jars, or glass jars. Only in 3 cases, white cotton t‐shirts have been chosen as stimuli collection material (Endevelt‐Shapira et al., 2018; Singh et al., 2018; Wintermann, Donix, Joraschky, Gerber, & Petrowski, 2013).

A wide spectrum of stimuli was assessed to induce emotion in the donors. Fear was evoked by watching horror video clips in 14 studies (Ackerl, Atzmueller, & Grammer, 2002; Chen, 2006; Chen & Haviland‐Jones, 2000; Ferreira et al., 2018; de Groot et al., 2012, 2018; de Groot, Semin, & Smeets, 2014a, 2014b; Groot, Smeets, Rowson, et al., 2015; Haviland‐Jones, McGuire, & Wilson, 2016; Iversen et al., 2015; Kamiloğlu et al., 2018; Zhou & Chen, 2009, 2011). In 10 studies, anxiety sweat was collected from students awaiting an oral examination at the university (Adolph, Meister, & Pause, 2013; Lübke, Busch, Hoenen, Schaal, & Pause, 2017; Pause, 2004a; Pause et al., 2009; Pause, Lübke, Laudien, & Ferstl, 2010; Prehn et al., 2006; Prehn‐Kristensen et al., 2009; Rocha et al., 2018; Wudarczyk et al., 2015, 2016).

In 7 studies, emotional response was elicited in donors by highly stressors events as first‐time tandem skydive (Endevelt‐Shapira et al., 2018; Mujica‐Parodi et al., 2009; Radulescu & Mujica‐Parodi, 2013; Rubin, Botanov, Hajcak, & Mujica‐Parodi, 2012) or high rope course (Albrecht et al., 2011; Haegler et al., 2010; Zernecke et al., 2011). The Trier social stress test (TSST), a validated protocol for inducing moderate levels of psychosocial stress, was administered to the donors in three studies (Dalton, Mauté, Jaén, & Wilson, 2013; Groot, Smeets, & Semin, 2015; Wintermann et al., 2013).

The competition was evaluated only by one study by collecting axillary sweat after an important badminton match (Adolph, Schlösser, Hawighorst, & Pause, 2010). Three studies evaluated the effect on receivers of sexual arousal induced by watching erotic video clips (Iversen et al., 2015; Zhou & Chen, 2011; Zhou et al., 2011). Four studies evaluated disgust evoked in donors by watching disgust‐evoking videos (Ferreira et al., 2018; de Groot et al., 2012; Iversen et al., 2015; Zheng et al., 2018).

In the majority of cases, the odor control condition was obtained by sweat pads collected after a neutral exercise session (e.g., ergometer trainings [Adolph et al., 2013; Albrecht et al., 2011; Haegler et al., 2010; Lübke et al., 2017; Mutic et al., 2016; Pause, 2004a; Prehn et al., 2006; Pause et al., 2009; Pause et al., 2010; Prehn‐Kristensen et al., 2009; Wintermann et al., 2013; Wudarczyk et al., 2015, 2016; Zernecke et al., 2011], treadmill exercise [Mujica‐Parodi et al., 2009; Radulescu & Mujica‐Parodi, 2013; Rubin et al., 2012], a running session [Adolph et al., 2010], stationary cycling [Dalton et al., 2013], hand‐bike training [Mutic et al., 2017], or nonstressed outdoor activity [Endevelt‐Shapira et al., 2018]).

Sweat pads collected after watching neutral videos (e.g., wildlife documentaries or weather forecasts) were used as body odor controls in 12 studies (Ackerl et al., 2002; Chen, 2006; de Groot, Semin, & Smeets, 2014a; de Groot, Semin, & Smeets, 2014b; Groot, Smeets, & Semin, 2015; Groot, Smeets, Rowson, et al., 2015; de Groot et al., 2018; Ferreira et al., 2018; Kamiloğlu et al., 2018; Zheng et al., 2018; Zhou et al., 2011; Zhou & Chen, 2011). Unused cotton pads were adopted by four research teams (Chen & Haviland‐Jones, 2000; de Groot et al., 2012; Iversen et al., 2015; Zhou & Chen, 2009).

In one study, control body odors were collected during an emotionally neutral situation (attending a regular class; Rocha et al., 2018).

In studies using tears as stimulus, sadness was evoked in female donors by watching sad films (Gelstein et al., 2011; Oh et al., 2012); the authors used as controls saline trickled down the cheek of donor women. In most studies, donors were tested to assess the right induction of the emotion during the experimental session. In some cases, a 7‐point Likert scale (Chen & Haviland‐Jones, 2000; Ferreira et al., 2018; de Groot et al., 2014a, 2014b; Groot, Smeets, Rowson, et al., 2015; Iversen et al., 2015; Kamiloğlu et al., 2018), a visual analog scale like the Positive And Negative Affect Schedule (PANAS; Chen, 2006; Mutic et al., 2017; Rocha et al., 2018; Wudarczyk et al., 2015; Zhou & Chen, 2009, 2011), or a self‐reported questionnaire (Dalton et al., 2013; Pause, 2004b; Prehn et al., 2006) was used.

In sixteen studies, a standardized validated scale measuring emotion was administered to donors, like the state‐trait anxiety inventory (Ackerl et al., 2002; Albrecht et al., 2011; D'Aniello et al., 2017; de Groot et al., 2012; Haegler et al., 2010; Lanata et al., 2018; Mujica‐Parodi et al., 2009; Radulescu & Mujica‐Parodi, 2013; Rocha et al., 2018; Zernecke et al., 2011), the Self‐assessment Manikin (SAM) (Adolph et al., 2013; Pause et al., 2009; Prehn‐Kristensen et al., 2009; Wintermann et al., 2013; Wudarczyk et al., 2015), or the State‐Trait Anger Expression Inventory (Mutic et al., 2016). Only in one case, the authors used a hidden camera to monitoring reactions associated with measurements of skin conductance, heart rate, and respiratory rhythm (Chen, 2006).

In order to assess stress reaction, salivary cortisol samples were collected in ten studies (Ackerl et al., 2002; Adolph et al., 2010; Groot, Smeets, & Semin, 2015; Endevelt‐Shapira et al., 2018; Mujica‐Parodi et al., 2009; Pause et al., 2009; Prehn‐Kristensen et al., 2009; Radulescu & Mujica‐Parodi, 2013; Rubin et al., 2012; Wudarczyk et al., 2016).

In all the analyzed studies, the receivers were healthy subjects with normal olfactory function and no respiratory diseases (age range 9–72 years); authors specified that recipient smokers were excluded in 19 studies. Receivers' olfaction was assessed by questionnaires or self‐reported in 10 papers (Ackerl et al., 2002; Chen, 2006; Dalton et al., 2013; Endevelt‐Shapira et al., 2018; Mujica‐Parodi et al., 2009; Pause et al., 2009; Prehn et al., 2006; Prehn‐Kristensen et al., 2009; Singh et al., 2018; Zheng et al., 2018), whereas smell threshold was assessed using the Sniffing' sticks test or its extended version (MONEX‐40) in 19 studies (Albrecht et al., 2011; Ferreira et al., 2018; de Groot et al., 2012, 2014a, 2014b, 2018; Groot, Smeets, Rowson, et al., 2015; Groot, Smeets, & Semin, 2015; Haegler et al., 2010; Iversen et al., 2015; Mutic et al., 2016, 2017; Rocha et al., 2018; Wintermann et al., 2013; Wudarczyk et al., 2015, 2016; Zernecke et al., 2011; Zhou & Chen, 2009; Zhou et al., 2011). Receivers were asked to identify phenylethyl alcohol in 6 papers (Adolph et al., 2010; de Groot et al., 2014a; Lübke et al., 2017; Pause et al., 2010; Zhou et al., 2011; Zhou & Chen, 2011), while the Brief‐Smell Identification Test (B‐SIT) was used in 1 paper (Zhou & Chen, 2011).

Stimulus was differently presented: Plastic or glass bottles were used as stimulus presentation tools in 3 early studies (Ackerl et al., 2002; Chen & Haviland‐Jones, 2000; Dalton et al., 2013); in the majority of subsequent studies, an olfactometer was used (Adolph et al., 2013; Adolph et al., 2010; Lübke et al., 2017; Mujica‐Parodi et al., 2009; Pause, 2004a; Pause et al., 2009; Pause et al., 2010; Prehn et al., 2006; Prehn‐Kristensen et al., 2009; Radulescu & Mujica‐Parodi, 2013; Rocha et al., 2018; Rubin et al., 2012; Wudarczyk et al., 2015; Zheng et al., 2018; Zhou et al., 2011); an intranasal Teflon tubing was used in one case (Wintermann et al., 2013). A band‐aid or a teabag attached just below the nostrils of receivers was used in 7 studies (Albrecht et al., 2011; Chen, 2006; Gelstein et al., 2011; Haegler et al., 2010; Oh et al., 2012; Wudarczyk et al., 2016; Zhou & Chen, 2009); as well in 7 studies, vials placed 2 cm below the participant's nose were used (de Groot et al., 2012, 2014a, 2014b; Groot, Smeets, Rowson, et al., 2015; Groot, Smeets, & Semin, 2015; Zernecke et al., 2011; Zhou & Chen, 2011); propylene jars were used in 4 cases (Ferreira et al., 2018; de Groot et al., 2018; Iversen et al., 2015; Kamiloğlu et al., 2018); a glass jar covered by a cap with an air filter was chosen by one research group (Endevelt‐Shapira et al., 2018). Cellulose filter mask or cotton pads in filter masks under the participants' noses were used in two papers (Mutic et al., 2016, 2017); a phantom patient wearing used cotton t‐shirts was selected as stimulus vehicle in one study as well (Singh et al., 2018).

Main outcomes were very heterogeneous too: Correct identification of the target emotion or odor rating was the main outcome of five studies (Ackerl et al., 2002; Chen & Haviland‐Jones, 2000; Haviland‐Jones et al., 2016; Iversen et al., 2015; Zhou & Chen, 2011). The influence of emotional chemosignals on cognitive tasks like performing word association while smelling one of the three types of olfactory stimuli was used by one research group (Chen, 2006). Priming of facial affect perception was the main outcome in one study (Pause, 2004a). Recognition of facial expressions after the exposition to anxiety or relaxed body odors was the main outcome in 4 papers (Mutic et al., 2016; Rocha et al., 2018; Zernecke et al., 2011; Zhou & Chen, 2009). The amplitude of the startle reflex recorded in the context of chemosensory anxiety signals was the main outcome in 4 studies (Adolph et al., 2013; Lübke et al., 2017; Pause et al., 2009; Prehn et al., 2006).

Amygdala activation during an fMRI session and ability to recognize ambiguous facial expression in relation to exposure to emotional stress body odors was used in one paper (Mujica‐Parodi et al., 2009). Brain areas activation after administration of chemosensory stimuli (Gelstein et al., 2011; Mutic et al., 2017; Prehn‐Kristensen et al., 2009; Radulescu & Mujica‐Parodi, 2013; Wintermann et al., 2013; Wudarczyk et al., 2015, 2016; Zheng et al., 2018; Zhou et al., 2011) as main outcome was analyzed in 9 studies. Haegler et al. investigated the risk‐taking behavior in computerized card games after smelling anxiety body odor (Haegler et al., 2010). Adolph et al. (2010) measured as main outcome skin conductance response of receivers in response to competition sweat. Authors investigated the influence of anxiety body odor on chemosensory event‐related potentials recorded during an EEG session in three studies (Adolph et al., 2013; Pause et al., 2010; Rubin et al., 2012). Measure of anxiety through the Spielberger's state‐trait anxiety inventory was evaluated in one study (Albrecht et al., 2011). In seven studies, authors investigated the ability to reproduce the same facial‐muscle configuration of the sender in the receiver with EMG (de Groot et al., 2012, 2014a, 2014b, 2018; Groot, Smeets, Rowson, et al., 2015; Groot, Smeets, & Semin, 2015; Kamiloğlu et al., 2018).

Singh et al. (2018) analyzed the effect of anxiety signals on the performance of dentistry students on three different dental procedures. Dalton and colleagues evaluated the influence of psychosocial stress body odor on social judgment (rating warmth and competence about women depicted in video scenario) (Dalton et al., 2013).

Appetite assessment by a visual analog scale (VAS) and food intake in men exposed to the smell of sad tears or trickled saline was the main outcome in 1 study (Oh et al., 2012). Cardiac parasympathetic activity measured in receivers was the main outcome in 1 case (Ferreira et al., 2018). Endevelt et al. evaluated autonomic and behavioral responses to social chemosignals in participants affected with autism spectrum disorder (Endevelt‐Shapira et al., 2018).

### 3.2 Interspecific communication

We found only three studies investigating the ability of animals to react to human chemosignals.

In 2016, for the first time in literature, Siniscalchi et al. tested the ability of 31 domestic dogs of various breeds (11 males and 20 females) to react to human chemosignals (Siniscalchi et al., 2016). Body odors stimuli of fear and joy were collected by 4 male donors, in whom emotions were elicited by watching comical or horror video clips; a 5‐point visual analogue scale and heart rate were examined to confirm the emotional response of the donors. Control stimuli were sweat pads collected after a nonstressful situation or after an exercise session. Main outcomes were dogs' cardiac activity and lateral asymmetry of dogs' nostril while sniffing different emotive stimuli.

Adopting an experimental paradigm based on behavioral responses on interhuman communication of emotions (de Groot et al., 2012), in the study by D'Aniello et al. (2018) 17 male and 23 female pet dogs (Labrador and Golden retrievers) were induced to smell “happy” and “fearful” human chemosignals collected from 8 male donors; the Spielberger's state‐trait anxiety inventory was used to control the emotion induction; unused sweat pads were employed as control stimuli; an odor container was located in a space where the dogs could move without restrictions. Authors analyzed the interactions of the dogs with their owner, with a stranger and with the experimental apparatus while sniffing different emotional body odors as main outcomes, dogs' stress, and heart rate were also measured.

Finally, after collecting human emotional body odors as in the previous study, Lanata et al. analyzed the Autonomic Nervous System reactions of 7 male horses in response to exposure to human happy and fearful chemosignals (Lanata et al., 2018). The main outcome was time‐frequency analysis of horses' heart rate variability.

## 4 DISCUSSION

The understanding of communication beyond words and body language is taking great interest; chemosignals transmitted through body odors may play a role in the communications in humans and between humans and other species.

The first peer‐reviewed article on this topic was published in 2000 by Chen and Haviland‐Jones (2000): The authors demonstrated that women performed better at olfactory identification of emotions than men, confirming previous data showing a better ability of women to recognize visual and auditory emotional signals (Brody & Hall, 2008).

Further studies confirm that women are better receivers for chemosignals than men (de Groot et al., 2014a); hence, the majority of the studies involves women as receivers and male as donors. It is clear that chemosignals from donors of the opposite sex are more effective than those from the same sex (Martins et al., 2005) pointing out that chemosignals may be important for reproductive purposes. On the other hand, there does not seem to be a different perception of chemosignals between different ethnicities, suggesting that chemosignaling communication could act beyond ethno‐cultural boundaries (de Groot et al., 2018).

A study on sexual appealing showed reduced physiological measures of arousal and lower levels of testosterone in men who sniffed tears from sad women compared to a control (Gelstein et al., 2011). Moreover, a study on the ability to react to body odors from partners demonstrated that intimacy enhances the detection of emotional cues, although not consciously (Zhou & Chen, 2011). Receivers are generally unable to consciously recognize the stimulus and name the body odor. On the other hand, this is not surprising, as olfaction has been termed “the mute sense” (Ackerman, 1991).

Several studies showed that humans, as well as animals, are influenced by the emotional state of other subjects, and that exposure to fear or anxiety‐related chemosignals can influence the performances of receivers in cognitive, behavioral, and emotional tasks (Adolph et al., 2013; Albrecht et al., 2011; Chen, 2006; de Groot et al., 2012, 2014a, 2014b; Groot, Smeets, & Semin, 2015; Ferreira et al., 2018; Kamiloğlu et al., 2018; Lübke et al., 2017; Mutic et al., 2016, 2017; Pause, 2004a; Prehn et al., 2006; Prehn‐Kristensen et al., 2009; Radulescu & Mujica‐Parodi, 2013; Rocha et al., 2018; Wudarczyk et al., 2015, 2016; Zernecke et al., 2011; Zhou & Chen, 2009; Zhou et al., 2011). Exposure to negative emotions heightened caution and vigilance in cognitive tasks (Chen, 2006), improved ability to recognize ambiguous faces expressions (Zernecke et al., 2011; Zhou & Chen, 2009), diminished the priming effect of happy faces in recognizing neutral faces (Mutic et al., 2016), and increased risk behavior in decision‐making tests (Haegler et al., 2010).

It has been suggested that increased perception and reaction to anxiety and fear may be responsible for social anxiety; in fact, Pause et al. demonstrated that the defense reflex and the required neuronal resources of anxiety‐related chemosignals were enhanced as in socially anxious receivers as compared to nonsocially anxious ones ( Pause et al., 2009; Pause et al., 2010).

Overall, negative emotions of the donor, as anxiety and fear, seem to be perceived by and influence social behavior in the recipient, inducing defense (Adolph et al., 2010), modifying risk‐taking behavior (Haegler et al., 2010), influencing performances in cognitive and perceptive tasks (de Groot & Smeets, 2017) by altering neuronal responses in the amygdala (Mujica‐Parodi et al., 2009), and in brain areas involved in the processing of emotions (Ackerl et al., 2002; Chen, 2006; Groot, Smeets, & Semin, 2015; Endevelt‐Shapira et al., 2018; Haviland‐Jones et al., 2016; Lübke et al., 2017; Pause, 2004a).

Chemical communication seems to be involved also in food choice and in the social importance of eating, having a huge impact in human social life, as demonstrated by Zheng et al. (2018): Body odors, collected after inducing disgust, activate social and emotional brain areas in recipients.

Even though negative emotions and sexual arousal have a more definite role in the human evolution, some evidences for the ability of humans to recognize and be influenced by the odor of happiness have been published (Chen & Haviland‐Jones, 2000; Groot, Smeets, Rowson, et al., 2015).

Data concerning the transmission of happiness have highlighted and extended the role of chemosignals in the interhuman communication, suggesting a more important role of these molecules other than the induction of the fight‐or‐flight response. Data on congenitally blinds individuals demonstrate an increased ability of these subjects, as compared to controls, to recognize chemosignals related to fear and disgust; on the other hand, blind subjects failed in identifying amusement and sexual body odors (Iversen et al., 2015). Taken together, these findings showed that negative emotions are better perceived by subject with impaired visual performance, suggesting an important role for the connection of vision and olfaction in identifying “positive” emotions, whereas negative emotions are well perceived by the sole use of olfaction. These observations underline the primitive role of olfaction in the fight‐or‐flight response.

The study of chemosignal communication may be important in psychiatric diseases as they could be useful in the diagnosis and maybe in the treatment of these diseases. On this regard, few studies have been published, namely on patients with panic disease (PD; Wintermann et al., 2013) and in patients with autism spectrum disorder (ASD; Endevelt‐Shapira et al., 2018). In patients with PD, brain areas involved in the process of anxiety chemosignals are altered (Wintermann et al., 2013) and this alteration may contribute in their panic response to environmental stimuli that are perceived as neutral for healthy individuals. Also in ASD, social anxiety chemosignals have different effect as respect to typically developed patients. Endevelt‐Shapira and colleagues showed a dissociated pattern of autonomic and behavioral responses in ASD subjects, suggesting a new interpretation to the impaired emotional regulation in ASD, whose underlying mechanisms are still unclear and can potentially open new perspectives of research for diagnosis and therapy of these patients (Endevelt‐Shapira et al., 2018).

In cognitively healthy subjects, anxiety chemosignals may influence job performances as it has been demonstrated by Singh et al.: In their experiment, authors showed that dentistry students worsened their professional performances if exposed to body odors produced in an anxiety‐inducing situation (Singh et al., 2018).

Notably in the majority of studies, the detection rate of the target emotion was very poor, suggesting that chemosignaling communication in humans acts below awareness (Pause et al., 2009; Zhou & Chen, 2009, 2011).

Chemosignals may also be important in mediating interspecific communication, especially in domesticated species such as dogs and horses living often in close proximity with humans. They have particular skills to detect and respond to human communicative signals, focusing mainly on gestures (D'Aniello, Scandurra, Alterisio, Valsecchi, & Prato‐Previde, 2016; Dorey, Conover, & Udell, 2014; Scandurra, Alterisio, Aria, Vernese, & D'Aniello, 2018; Scandurra et al., 2017).

Dogs and horses went through convergent evolution, whereby they have become human social partners, in which the reciprocal reading of the emotional status would be a very useful tool in many situations and has an important biological fitness benefit. Indeed, dogs and horses are able to recognize and appropriately respond to human emotions by interpreting visual and acoustic messages (Albuquerque et al., 2016; Merola, Marshall‐Pescini, D'Aniello, & Prato‐Previde, 2013; Morisaki, Takaoka, & Fujita, 2009; Nagasawa, Murai, Mogi, & Kikusui, 2011; Smith, Proops, Grounds, Wathan, & McComb, 2016). However, such species are much more olfactory focused than humans, which make them excellent study models for researches on chemosignaling. Results showed that human fear chemosignals induced the reproduction of behaviors and physiological state of the sender in dogs (D'Aniello et al., 2018; Siniscalchi et al., 2016). Moreover, dogs exposed to human happiness chemosignals appeared more confident with strangers, implying that a relaxed mood of owners calms their pet dogs (D'Aniello et al., 2018). In horses, human fear and happiness chemosignals induced sympathetic and parasympathetic changes indicating emotional activation (Lanata et al., 2018). However, this latter study, while providing interesting data, remains preliminary, due to the little sample size.

Overall fear, anxiety, dominance, and sexual arousal are the most recognized emotions through chemosignals (de Groot & Smeets, 2017), whereas the demonstration of recognition of happiness is less frequent (Groot, Smeets, Rowson, et al., 2015). This was also true in humans if the pattern of emotional recognition used is visual (Jiang, Costello, Fang, Huang, & He, 2006; Pourtois, Grandjean, Sander, & Vuilleumier, 2004), which make the data less robust and awaiting confirmation. Alternatively, it is possible that emotions such as fear, anxiety, dominance, and sexual arousal could be more easily recognized in contrast to happiness, due to their major evolutionary relevance and reproductive role.

## 5 CONCLUSIONS

Despite the wide heterogeneity between studies and the small sample sizes analyzed, the evidences highlight the importance of chemosignals in social interaction, empathy with the partner, social judgment, danger detection, social aspect of eating, risk‐taking behavior, stressful performance, and perhaps perception of happiness. Less evidence of a role of chemosignals in personality disorders and psychiatric pathologies is available, and there are no data on chemosignaling neurodegenerative and age‐related brain diseases. Improving our knowledge on chemosignal communication in patients with psychiatric or neurodegenerative disorders could be of paramount importance to better understand the disease pathophysiology and to develop new diagnostic and therapeutic strategies, and to this extent, the adoption of a clear evidence‐based study design is of fundamental importance.

## CONFLICT OF INTEREST

The authors have no conflicts of interest to declare.

## AUTHOR CONTRIBUTION

PDA and BDA conceived and supervised the study, AS and MM supervised the study, EC and UQ retrieved the data, and all the authors wrote and approved the manuscript.

## Data Availability

Data sharing is not applicable to this article as no new data were created or analyzed in this study.
